# Long-term efficacy of cyclosporine and interferon-ω in feline chronic gingivostomatitis: insights from SDAI scores

**DOI:** 10.1186/s12917-025-05141-9

**Published:** 2025-12-16

**Authors:** Kue Hwan Choe, Kwangsik Jang, Se Eun Kim, Hyun Min Jo

**Affiliations:** 1 Taeil animal dental clinics, Seoul, 01394 Republic of Korea; 2 Department of Veterinary surgery, College of Veterinary Medicine, BK21 Plus Project Team, Chonnam National University, Gwangju, 61186 Republic of Korea; 3 Biomaterial R&BD Center, Chonnam National University, Gwangju, 61186 Republic of Korea

**Keywords:** Cyclosporine, Feline chronic gingivostomatitis, Interferon-ω, Stomatitis disease activity index

## Abstract

**Background:**

Feline chronic gingivostomatitis (FCGS) is a challenging disease with unclear causes and limited treatment options. The most effective treatment for FCGS is tooth extraction. However, most cats require additional drug treatment for refractory FCGS even after undergoing extractions. We hypothesized that the combination of interferon-ω (IFN) and cyclosporine (CsA) would improve clinical outcomes. This study aimed to evaluate the long-term efficacy of IFN and CsA in FCGS using Stomatitis Disease Activity Index (SDAI) scores. In this study, the treatment process was divided into two stages: in the surgical stage (Stage 1), all cats underwent tooth extraction combined with submucosal IFN injection; in the immunomodulatory stage (Stage 2), additional IFN/CsA treatment was administered to cats with refractory FCGS.

**Results:**

The therapeutic response was monitored during treatment period using SDAI scores. The patients were classified into four groups by oral examination: Group A (Surgical remission), Group B (Substantial improvement to resolution), Group C (Partial improvement), and Group D (No response). Patients achieving complete remission following surgical treatment were classified into Group A (39.2% of all cases). Patients assessed as having refractory FCGS after surgical treatment accounted for 60.8% of all patients and were divided into three groups. Groups B, C, and D accounted for 52.1%, 45.2%, and 2.7% of refractory cases, respectively. In addition, the SDAI scores showed significant differences across groups at all time points. Receiver operating characteristic curve analyses identified key SDAI thresholds for early classification: a T_1_ score cutoff of 6.5 and a T_0_–T_1_ score change cutoff of − 14.5 effectively distinguished Group A. The long-term follow-up revealed that cats classified as “subs-resolution” at T_6_ were significantly more likely to sustain clinical improvement for ≥ 2 years than those in the “non-partial” group.

**Conclusion:**

This study showed that the combination of IFN and CsA is an effective therapeutic option for refractory FCGS, with more than half of treated cats showing clinical improvement and 76.5% of those achieving subs–resolution at 6 months maintaining remission for 2–6 years. These findings further indicate that evaluating SDAI scores six months after a three-month course of immunomodulatory therapy provides prognostic value for predicting long-term outcomes.

**Supplementary Information:**

The online version contains supplementary material available at 10.1186/s12917-025-05141-9.

## Background

Feline chronic gingivostomatitis (FCGS) is a painful and often debilitating disease in cats characterized by protracted oral inflammation that typically lasts months to years [1]. This condition is an immune-mediated oral mucosal inflammatory disease of cats with a reported prevalence of 0.7–26% in the cat population [1–5]. FCGS affects the oral mucosa of cats and is presented in two clinical phenotypes: ulcerative and proliferative. Some cats may exhibit both clinical manifestations simultaneously [6, 7]. FCGS lesions are typically associated with moderate to severe oral pain, and in some cases, the condition can lead to significant discomfort and potentially life-threatening consequences in approximately 10% of affected cats [7, 8]. Clinical signs often include reduced appetite, decreased grooming, and diminished social behavior [9–11].

The etiology of FCGS is characterized by an inappropriate immune response chronic antigenic stimulation within the dental and oral environment, with proposed contributing factors including viral infection, bacterial involvement, and dysbiosis of the oral microbiome; however, the exact cause remains unclear [12–15]. This inadequate immune response is characterized by exaggerated and chronic lymphoplasmacytic inflammation driven by dysregulated T-cell activity and pro-inflammatory cytokine imbalance, resulting in persistent mucosal inflammation unresponsive to conventional anti-inflammatory therapy. In general, immune-mediated diseases result from an imbalance in T-cell subsets and inflammatory cytokines and lead to chronic inflammatory conditions [16]. In FCGS, current etiologic theories suggest the involvement of a CD8⁺ T-cell-mediated immune response, chronic infection with feline calicivirus (FCV), increased expression of pro-inflammatory cytokines such as IL-6 and IL-17, and the activation of pathways associated with myeloid-lineage cells of the innate immune system [11, 17–19]. Furthermore, coinfections with FCV, feline foamy virus (FFV), or feline leukemia virus (FeLV) have been linked to poorer treatment outcomes and increased disease severity [19, 20].

There is currently no definitive treatment for FCGS. The standard approach involves partial-mouth extraction (PME; extraction of premolar and molar teeth) or full-mouth extractions (FMEs) with or without the use of supportive treatments such as antibiotics, analgesics, and anti-inflammatory medications. Such medical treatments may need to be extended for weeks or months when cats are not sufficiently improved after dental extractions; this has been called extended medical management (EMM) [10]. Additional therapies may include immunomodulatory agents such as recombinant feline interferon-ω (IFN), cyclosporine (CsA), or mesenchymal stem cell (MSC) therapy [1, 7, 21]. In addition, corticosteroids such as prednisone have also been used; however, due to their limited long-term efficacy and the risk of adverse effects including polyuria, polydipsia, diabetes mellitus, and skin fragility, they are no longer considered a preferred option and are now recommended only for short-term symptomatic relief [1, 6, 7, 22]. Moreover, nonsteroidal anti-inflammatory drugs (NSAIDs) are also used as an alternative to corticosteroids for controlling pain and inflammation in FCGS [20]. Among those who have undergone tooth extraction and EMM, some show improvement, while others do not. In previous research, 21% of patients improve without medication after tooth extraction, while 46.3% show improvement with medical therapy following tooth extraction [10]. However, 32.6% of cats do not respond to tooth extraction and EMM. In some cases, the persistent and severe clinical manifestations of refractory FCGS—such as severe oral pain, halitosis, ptyalism, weight loss, and decreased activity—may significantly impair the cat’s quality of life to the extent that humane euthanasia is ultimately considered [1, 7, 8].

Some cats may fail to achieve significant clinical improvement following dental extractions and initial medical treatment and are considered refractory cases. The definition of “refractory cases” varies among specialists; however, refractory FCGS refers to a condition where the lesions of FCGS have persisted for a certain period after tooth extraction and initial medical treatment and are associated with significant clinical signs such as pain and anorexia. Depending on the authors, the time delay to consider the case “refractory” varies between 28 and 50 days post-extraction [7, 10, 11, 21, 22]. For these refractory cases, adjunct immunomodulatory treatments such recombinant feline IFN, CsA and, MSC therapy have been proposed and may result in different outcomes [21–25]. Topical application of IFN to oral lesions once daily has been found to lead to clinical improvement in 55% (12/22) of the cases [22]. In addition, during a 6-week treatment period with CsA, 77.8% of the treatment group showed an improvement of more than 40% in Stomatitis Disease Activity Index (SDAI) scores compared with before treatment, and 5 of the 11 cats (45.5%) were clinically cured (defined as having no residual oral inflammation) after having received CsA for 3 months or more [21]. MSC therapy has been actively investigated in recent years as a promising treatment for refractory FCGS. In earlier studies, autologous MSC treatment resulted in clinical improvement in 5 of 7 cats (71.4%), while allogeneic MSC treatment led to improvement in 4 of 7 cats (57%) [24, 26]. More recently, a long-term evaluation of 29 cats (2–9 years), clinical remission was achieved in 17 of 29 cats (58.6%), including 9 of 14 cats (64%) treated with autologous MSCs and 8 of 15 cats (53%) treated with allogeneic MSCs [8].

The SDAI is a scoring system that quantifies the severity of FCGS by incorporating factors such as changes in body weight, owner-reported assessments, and the degree of inflammation observed at various oral lesion sites. This system has been widely used for clinical evaluation of FCGS over time. Prior to the implementation of such structured assessments, disease progression was evaluated primarily based on the subjective impressions of veterinarians and cat owners. The adoption of the SDAI has enabled more standardized and consistent comparisons of clinical improvement.

Although CsA and IFN have been investigated to treat refractory FCGS, their combined use and its potential to induce lasting remission has not been studied. This study aimed to assess the combined therapeutic effects of IFN and CsA used after tooth extraction. This retrospective analysis included long-term follow-up data (up to six years) from cats treated with this combination therapy. The findings may support the long-term efficacy of immunomodulatory treatment in FCGS and contribute to improved understanding of treatment outcomes in refractory cases.

## Methods

### Case inclusion

Cats presenting with ulcerative or proliferative lesions in the caudal oral mucosa were diagnosed with FCGS. A total of 220 cats diagnosed with FCGS visited the Taeil Veterinary Dental Hospital between 2018 and 2023. Among them, cats were excluded from the study if they did not undergo consistent follow-up after extractions due to various reasons including owner refusal of either scheduled rechecks after surgery or further treatment following a diagnosis of refractory FCGS or failure to administer the prescribed immunomodulatory medications (IFN/CsA) during the treatment period. In addition, cats with FCGS lesion isolated to regions other than the caudal oral mucosa were excluded because lesion severity and treatment response could not be consistently evaluated during the treatment process. As a result, 120 cats met the inclusion criteria for this study.

All 120 cats underwent pre-anesthetic blood tests, thoracic radiographic evaluations, and pathogen screening. Heartworm, feline immunodeficiency virus (FIV), and FeLV statuses were tested using ELISA on blood samples. FCV, feline herpesvirus (FHV), and *Mycoplasma* spp. were tested for using PCR on oral mucosal swabs. These screenings were conducted to evaluate each cat’s systemic health and immune status before treatment. Positive results were recorded descriptively but were not used as exclusion criteria, provided the cats were clinically stable and free of systemic illness.

### Overall treatment process for FCGS

#### Surgical procedure, postoperative management, and outcome criteria (Stage 1)

Cats presenting with ulcerative or proliferative lesions in the caudal oral mucosa were diagnosed with FCGS, and teeth with inflammatory lesions in the surrounding gingiva were extracted. Depending on the severity and location of the lesions, a partial-mouth extraction (PME), which included all of the teeth caudal to the canines, or a FME was performed. All cats received 1.5 million units (MU) of IFN (Virbagen Omega^®^, Virbac, Carros, France) via submucosal injection into each caudal oral mucosal area during the dental extraction procedure.

All cats were postoperatively treated with antibiotics for 14 days; either amoxicillin-clavulanate (12.5 mg/kg PO BID) or cefovecin (8 mg/kg SC) was used. For pain management, gabapentin (5 mg/kg PO BID) was prescribed for two weeks, and a transdermal fentanyl patch was applied immediately after surgery for acute postoperative analgesia. Two weeks after surgery, additional analgesics such as gabapentin or opioids were administered as needed during the Stage 1 evaluation period.

The period between 4- and 12-weeks post-extraction (extendable up to a maximum of 6 months) was defined as the Stage 1 evaluation period for determining surgical remission or refractory FCGS.

**Fig. 1:**
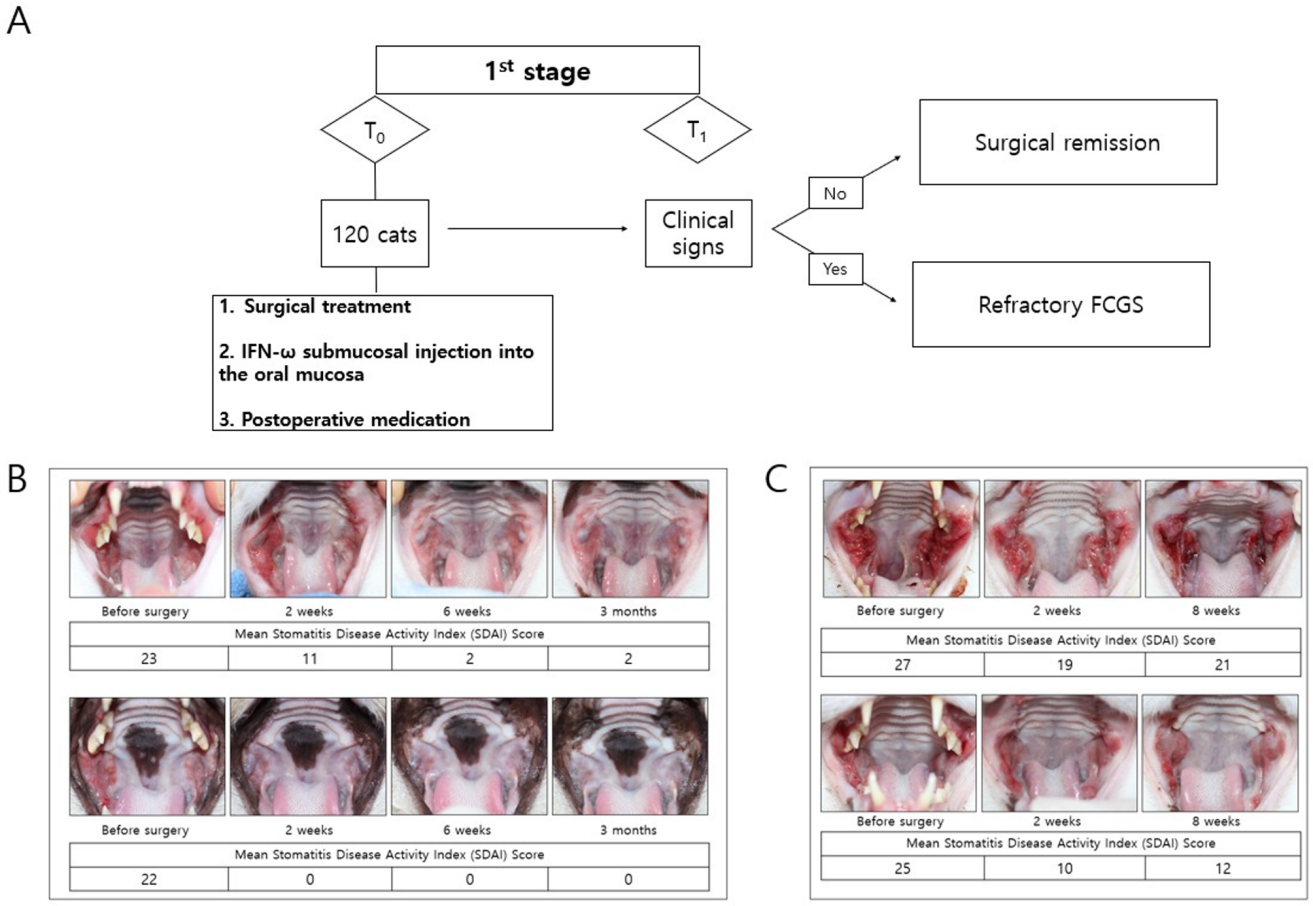
Stage 1 outcome evaluation and classification following dental extractions in cats with FCGS. **(A)** Flowchart summarizing the Stage 1 evaluation process. A total of 120 cats diagnosed with FCGS underwent dental extractions and received submucosal IFN injections and postoperative medication. Surgical remission was defined as the resolution of clinical signs without further medical treatment, while the presence of persistent or recurrent clinical signs was classified as refractory FCGS. **(B)** Representative oral images of cats classified as surgical remission cases. Clinical signs and oral lesions resolved without the need for immunomodulatory therapy. The SDAI scores and corresponding percentage improvement are shown below each timepoint. **(C)** Representative oral images of cats classified as refractory FCGS. Despite initial improvement, clinical signs and lesions persisted or recurred, requiring further treatment. The SDAI scores and percentage improvement are shown for each timepoint

Surgical remission was defined as the resolution of clinical signs without the need for additional medical treatment. In cases where oral lesions persisted despite the resolution of clinical signs, the observation period was extended up to six months. If no recurrence of clinical signs was observed during this period and the oral lesions showed gradual improvement, the case was ultimately classified as surgical remission. In contrast, cases were diagnosed as refractory FCGS if the clinical signs recurred and the lesions worsened during the observation period or if the clinical signs persisted and the oral lesions remained unchanged, worsened, or showed only minimal improvement (Fig. 1).

#### Immunomodulatory treatment, monitoring, and outcome criteria (Stage 2)

Patients diagnosed with refractory stomatitis were treated with additional immunomodulatory therapy (IFN/CsA) (Stage 2). Oral mucosal IFN was administered via topically at a dose of 0.1 MU once daily. This application was performed at home by the owners according to veterinary instructions, which included positioning the syringe caudally to the canines in the oral commissure under the cheek, slowly dispensing the solution while keeping the cat’s mouth closed for 30 s to 1 min to maximize mucosal contact and prevent spillage. The initial dose of CsA (Sandimmune^®^, Novartis, Basel, Switzerland) was 5 mg/kg PO BID. Both medications were administered for a duration of three months.

After three months, if the oral lesions had significantly improved and the clinical signs had resolved, tapering of the medication was initiated with the goal of complete discontinuation. In cases showing only a minimal response, tapering was initiated with careful monitoring for recurrence. When tapering or discontinuation was attempted, repeated follow-up evaluations were conducted within one to two months to assess for recurrence. If the lesions relapsed or worsened, medical treatment was resumed.

Compliance with the immunomodulatory treatment was assessed through regular consultations with the owners during the treatment period. To verify adherence, the remaining amount of medication was measured at each follow-up visit to ensure that it was being administered as prescribed. In addition to compliance, treatment monitoring during the immunomodulatory period included regular follow-up visits to assess each patient’s clinical signs, body weight, appetite, and general activity. When clinically indicated, diagnostic testing such as bloodwork (complete blood count and serum biochemistry) and thoracic radiographs were performed to detect potential subclinical complications associated with prolonged CsA administration.

**Fig. 2:**
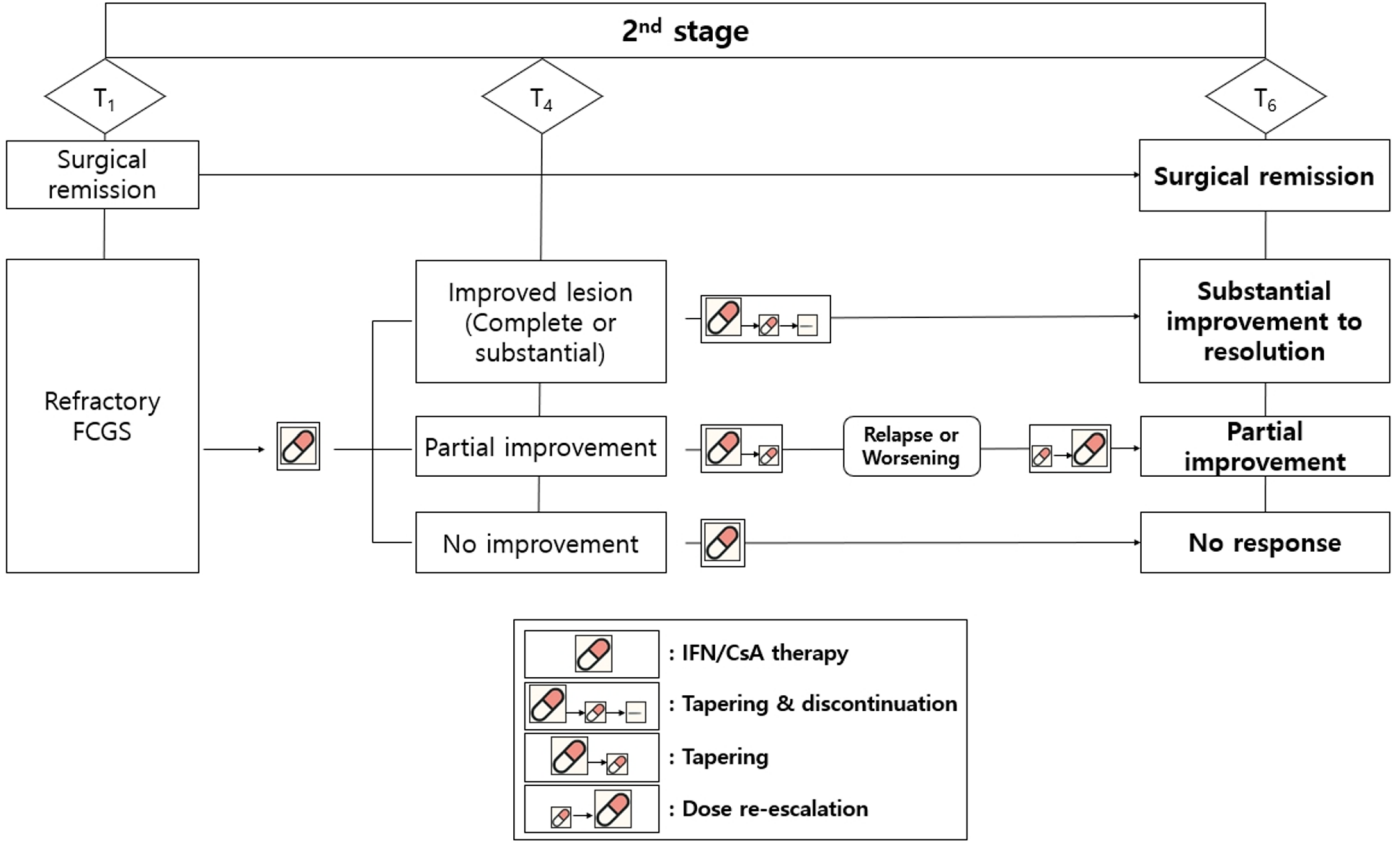
Stage 2 treatment flow and classification of long-term outcomes in refractory FCGS cats

The recheck timepoint was set at approximately 6 months (mean 169 days, range 130–209 days) after the end of the 3-month immunomodulatory treatment and was used to classify treatment outcomes. These included the following: [1] cats that had surgical remission without requiring immunomodulatory therapy; [2] cats that achieved substantial improvement or resolution after immunomodulatory treatment discontinuation; [3] cats that showed partial improvement but experienced recurrence after discontinuing immunomodulatory therapy; and [4] cats that showed no response to immunomodulatory therapy (Fig. 2).

The severity of the lesions and prognosis were assessed using the SDAI scoring systems by a single veterinarian.

Cats that did not achieve remission during Stage 1 received immunomodulatory therapy (IFN/CsA) from T_1_ to T_4_. At T_4_, the treatment response was categorized as improved (complete or substantial improvement), partial improvement, or no improvement. Depending on clinical progression, tapering, discontinuation, or dose re-escalation of the medication was performed. The final outcomes were assessed at T_6_ timepoint and classified as follows:

 [1] Surgical remission,

 [2] Substantial improvement to resolution after treatment discontinuation,

 [3] Partial improvement without successful discontinuation, and.

 [4] No response to immunomodulatory therapy.

Treatment modifications are indicated by the icons in the legend: IFN/CsA therapy, tapering and discontinuation, tapering only, and dose re-escalation.

### Global classification of the outcome according to the oral examination

**Fig. 3:**
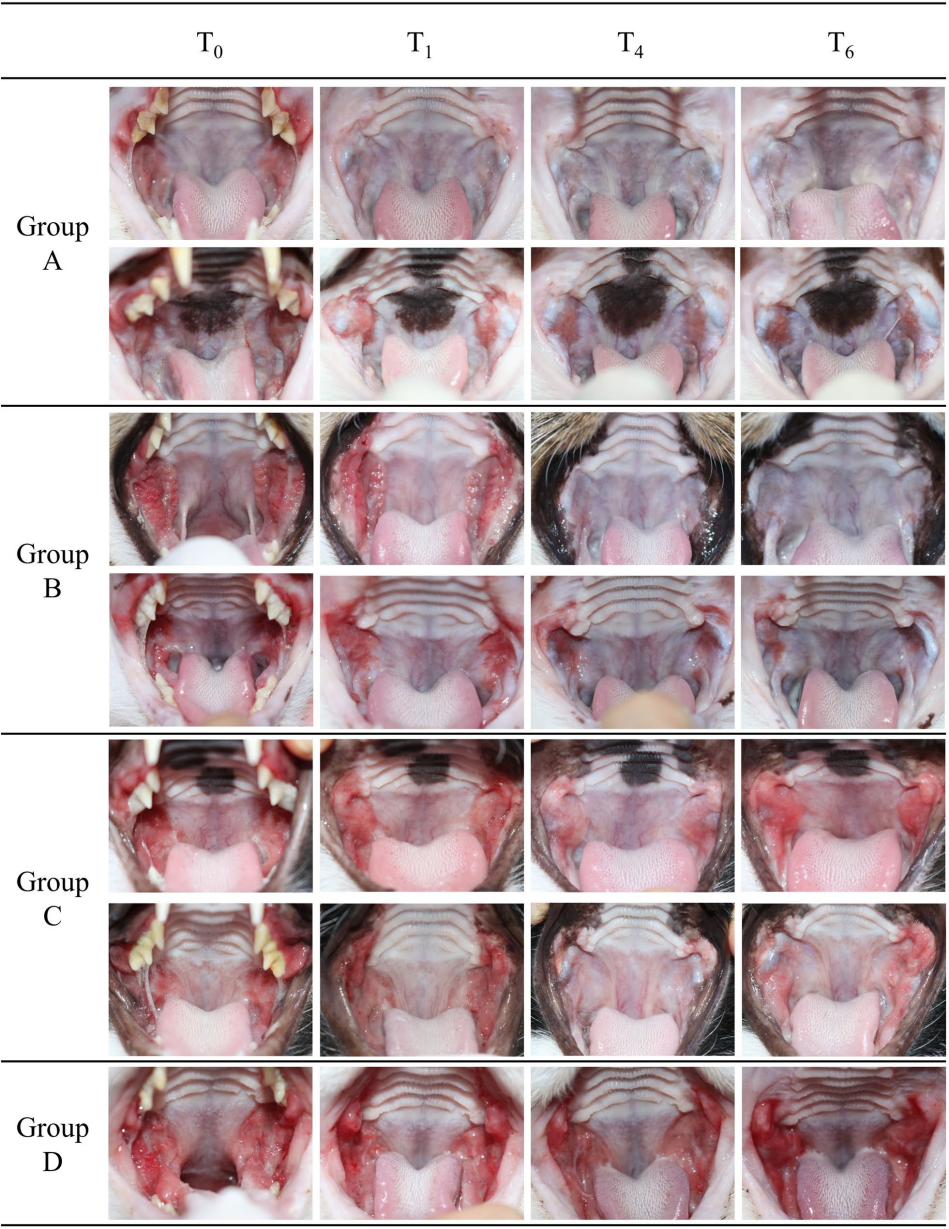
Group classification of patient according to the oral examination

All cats were categorized into four groups at the time of T_6_ based on their overall treatment outcomes, reflecting the combined effects of surgical treatment (tooth extraction with submucosal injection of IFN) and the presence or absence of subsequent immunomodulatory treatment (IFN/CsA). (Fig. 3).

Group A (Surgical remission):

This group included cats with lesions that improved following surgical treatment, which involved tooth extraction combined with a single submucosal injection of IFN into the caudal oral mucosa, without requiring additional immunomodulatory treatment (IFN/CsA). These cats no longer required further treatment.

Group B (Substantial improvement to resolution):

This group included cats that did not sufficiently respond to surgical treatment and continued to show clinical signs, achieving either complete or substantial improvement but persistent remission only after the subsequent administration of immunomodulatory treatment (IFN/CsA), which was gradually tapered and discontinued without lesion relapses.

Group C (Partial improvement):

This group consisted of cats that showed only partial improvement. Tapering of IFN and/or CsA was attempted—typically by reducing the dosing frequency to every other day or twice weekly over a period of two to four weeks. However, most cats experienced relapses or worsening of oral lesions following tapering, necessitating the resumption of continuous therapy.

Group D (No response):

This group included cats that showed no improvement or worsening despite continued immunomodulatory treatment following surgical treatment; they experienced persistent oral lesions and clinical signs.

In Group A, there was no additional medical treatment for FCGS other than surgical extractions and intralesional injection of IFN into the oral mucosa. In Group B, additional immunomodulatory treatment for FCGS was administered after surgical extractions, resulting in substantial to resolution of lesions. No recurrence of the lesions was observed after discontinuation of the medication. In Group C, immunomodulatory treatment continued until the oral erythema no longer improved. After discontinuing the medication, patients returned within one to three months, and the previously improved oral lesions had worsened again. In Group D, no improvement in oral lesions was observed despite additional immunomodulatory treatment.

Group A: Surgical remission; Group B: Substantial improvement to resolution; Group C: Partial improvement; Group D: No response; T_0_: before extraction; T_1_: start of IFN/CsA treatment; T_4_: 12 weeks after IFN/CsA; T_6_: 6 months after T_4_.

### SDAI scoring system

The SDAI scoring system used in this study was a modified version of the original index developed by Jamie Anderson [8, 21, 24–26]. It consisted of ten components, each scored on a scale from 0 to 3, for a total of 30 points (Figure S1). A lower total score indicated minimal or no disease activity, while a higher score reflected more severe clinical symptoms. First, a composite owner-reported score was included, derived by averaging the ratings for four behavioral parameters: appetite, activity level, grooming, and perceived oral comfort. Second, body weight change was also evaluated as an independent item. The remaining eight components assessed the degree of inflammation at eight oral sites: the maxillary- and mandibular-attached gingiva and buccal mucosa, sublingual/lingual area, palatoglossal arch, molar salivary gland, and oropharyngeal region.

The SDAI scores were evaluated for all cats at different timepoints: T_0_ (before dental extractions), T_1_ (start of immunomodulatory (IFN/CsA) treatment), T_2_ (4 weeks after treatment), T_3_ (8 weeks after treatment), and T_4_ (12 weeks after treatment), T_6_ (over 6 months after T_4_). At the T_6_ timepoint, cats were classified into four prognostic groups based on treatment outcomes. Differences in SDAI score changes among these groups were statistically analyzed at each timepoint. To ensure consistency in evaluation, all SDAI scores were assessed and recorded by a single veterinarian. In cases where cats were uncooperative or exhibited oral pain, sedation was administered either at home prior to the visit or at the hospital before the examination.

### Long-term outcome classification

For the purpose of long-term prognostic analysis, additional subgroup terms were used in a subset of cats that completed the initial treatment and were available for follow-up of ≥ 2 years. Cats showing substantial improvement or resolution at T_6_, corresponding to a portion of Group B, were designated as the “Subs-resolution” group. Conversely, cats showing partial or no improvement at T_6_, corresponding to subsets of Groups C and D, were categorized as the “non-partial” group. These subgroup terms were used exclusively for statistical comparisons in the long-term outcome analysis and do not represent new clinical classifications.

### Statistical analysis

The Kruskal–Wallis test was used to evaluate differences in SDAI scores among groups because the data were not normally distributed. To account for preoperative differences, Quade’s nonparametric ANCOVA was applied. For post-hoc comparisons, pairwise tests and additional analyses of median differences between groups were performed using Bonferroni correction. In addition, receiver operating characteristic (ROC) curve analyses were conducted to evaluate the discriminatory ability of SDAI scores and score changes in classifying treatment outcome. In particular, separate ROC curve analyses were performed to distinguish cases with surgical remission (Group A) from refractory cases (Group B, C, D) using the SDAI score at T_1_ and the change in SDAI scores from T_0_ to T_1_.

To further evaluate long-term outcomes, logistic regression analyses were performed to assess the association between T_6_ classifications (“Subs-resolution” (Substantial improvement to resolution) vs. “Non-partial” (No response and Partial improvement) and sustained clinical improvement for ≥ 2 years. Odds ratios (ORs) with 95% confidence intervals (CIs) were calculated to quantify the predictive value of T_6_ response status.

## Results

### Patient demographics and infectious disease seroprevalence among 120 cats

Among the 120 cats included in the study, the mean age at the time of tooth extraction was 5.9 years (range: 1–15 years). Additional patient characteristics are summarized in Table 1.

**Table 1:** Seropositivity for infectious diseases and signalment of 120 cats with FCGS

Variable	Positive No. (%) of cats
Infectious agent	
Feline calicivirus	106/120 (88.3)
Herpesvirus	2/120 (1.7)
Mycoplasma	17/120 (14.2)
Feline immunodeficiency virus	2/120 (1.7)
Feline leukemia virus	1/120 (0.8)
Heartworm	0/120 (0)
Sex	
Intact female	2/120 (1.7)
Spayed female	67/120 (55.8)
Intact male	2/120 (1.7)
Castrated male	49/120 (40.8)
Breed	
Domestic Shorthair	111/120 (92.5)
Turkish Angora	6/120 (5.0)
Norwegian Forest Cat	2/120 (1.7)
Russian Blue	1/120 (0.8)

### Group distribution among 120 cats

To better understand treatment outcomes, we grouped the cats into four broader categories based on their oral examination results. Group A, which included cats that complete remission following tooth extraction with a single submucosal injection of IFN, had the highest number of cats, with 47 individuals (39.2%). Surgical treatment did not completely resolve the lesions in 73 cats (60.8%), which were considered to have refractory FCGS. Group B, which included cats with substantial improvement to resolution following a combination of surgery and immunomodulatory therapy, consisted of 38 cats (52.1%). Group C, representing cats with partial improvement, included 33 cases (45.2%), while Group D, which included cats with no response to treatment, had the fewest, with 2 cats (2.7%) (Table 2). Together, Groups A and B (representing cats with substantial improvement to complete remission achieved through surgical and/or immunomodulatory treatment) accounted for 85 cats (70.9% of all cases) (Table 2).

**Table 2:** Group distribution in the treatment of FCGS in 120 cats

Stage 1	No. of cats (%)	Stage 2	No. of cats (%)
Group A	47/120 (39.2)	N/A	N/A
Refractory FCGS	73/120 (60.8)	Group B	38/73 (52.1)
		Group C	33/73 (45.2)
		Group D	2/73 (2.7)

Group A: Surgical Remission; Group B: Substantial improvement to resolution with immunomodulatory treatment (IFN/CsA); Group C: Partial Improvement with immunomodulatory treatment; Group D: No Response

### Distribution of SDAI scores for 120 cats at each timepoint

The SDAI scores for each group showed statistically significant differences at every timepoint (Table 3). At T_0_, before any treatment, the median SDAI scores for Groups A, B, C, and D were 23.00, 24.50, 27.00, and 27.50 respectively, and there were statistically significant differences between Groups A and C (*p* < 0.01).

**Table 3:** SDAI score distribution for each group of 120 cats with FCGS

Variation	Category	Median	Interquartile Range	H	*p*-value	Post hoc validation
T_0_	Group A	23.00	6.00	28.697	< 0.01	C > A
	Group B	24.50	3.00			
	Group C	27.00	4.50			
	Group D	27.50	.			
T_1_	Group A	2.00	2.00	41.417	< 0.01	B, C > A
	Group B	17.00	8.00			
	Group C	19.00	7.00			
	Group D	15.50	.			
T_2_	Group A	0.00	2.00	29.751	< 0.01	D > A B, C > A
	Group B	7.00	6.50			
	Group C	10.00	9.00			
	Group D	14.00	.			
T_3_	Group A	0.00	2.00	14.583	< 0.01	D > A C > B > A
	Group B	2.00	5.00			
	Group C	7.00	8.00			
	Group D	14.50	.			
T_4_	Group A	0.00	2.00	10.306	< 0.01	D > A, B C > A, B
	Group B	1.00	2.00			
	Group C	5.00	9.00			
	Group D	14.50	.			
T_6_	Group A	0.00	2.00	10.862	< 0.01	D > A, B C > A, B
	Group B	0.00	1.00			
	Group C	3.00	10.00			
	Group D	19.50	.			

Group A: Surgical remission; Group B: Substantial improvement to resolution with immunomodulatory treatment (IFN/CsA); Group C: Partial improvement with immunomodulatory treatment; Group D: No response; T_0_: before extraction; T_1_: start of immunomodulatory treatment; T_2_: 4 weeks after immunomodulatory treatment; T_3_: 8 weeks after immunomodulatory treatment; T_4_: 12 weeks after immunomodulatory treatment; T_6_: 6 months after T_4_; “.” indicates that the interquartile range was 0 and omitted for simplicity

At T_1_, a sharp reduction in scores was observed in Group A, while Groups B, C, and D maintained relatively higher scores. Group A differed significantly from all others, indicating early surgical response.

From T_2_ to T_4_, a gradual decline in scores was noted in Groups B and C, while Group D showed little change. Group A consistently maintained the lowest scores. Post hoc comparisons revealed significant differences between Group A and all others at each timepoint, and between Groups B and C at T_3_ and T_4_.

By the final evaluation (T6), both Groups A and B reached zero median SDAI scores, whereas Group C had median of 3, and Group D retained higher scores. Both Groups C and D showed statistically significantly higher SDAI scores than Groups A and B (*p* < 0.01); notably, Group D had the highest residual disease activity.

**Fig. 4:**
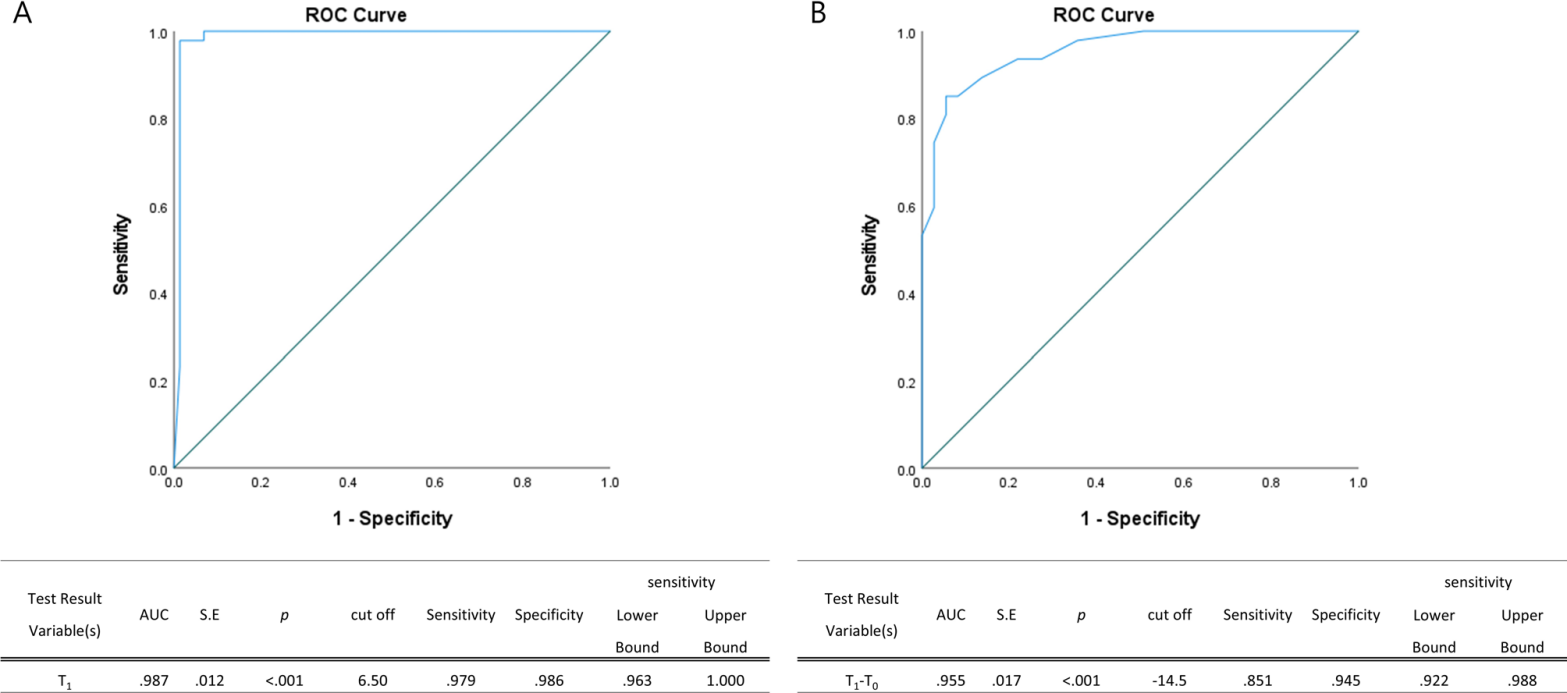
ROC curves classifying of surgical remission and refractory FCGS cases based on the SDAI score. The ROC curves illustrate the classification performance based on the score at T_1_ (**A**) and the change in score from baseline to T_1_ (**B**) in distinguishing surgical remission from refractory FCGS cases. A cutoff value of 6.50 at T_1_ yielded a sensitivity of 97.9% and specificity of 98.6% (AUC = 0.987, 95% CI: 0.963–1.000; *p* < 0.001). For the change in score (T_1_–T_0_), a cutoff of − 14.5 provided a sensitivity of 85.1% and specificity of 94.5% (AUC = 0.955, 95% CI: 0.922–0.988; *p* < 0.001). Group A: Surgical Remission; T_0_: before extraction T_1_: start of IFN/CsA treatment.

To further assess the utility of the SDAI in classifying treatment response, ROC curve analyses were performed (Fig. 4). The SDAI score at T_1_ distinguished Group A from refractory cases (Groups B, C and, D) with excellent accuracy (cutoff value = 6.5, sensitivity 97.9%, specificity 98.6%, area under the curve (AUC) = 0.987, 95% confidence interval (CI): 0.963–1.000, *p* < 0.001; Fig. 4A). The change in the SDAI score from T_0_ to T_1_ also showed high discriminative power (cutoff value = −14.5, sensitivity 85.1%, specificity 94.5%, AUC = 0.955, 95% CI: 0.922–0.988, *p* < 0.001; Fig. 4B).

### Long-term clinical outcomes (≥ 2 years) by T_6_ classification

**Fig. 5:**
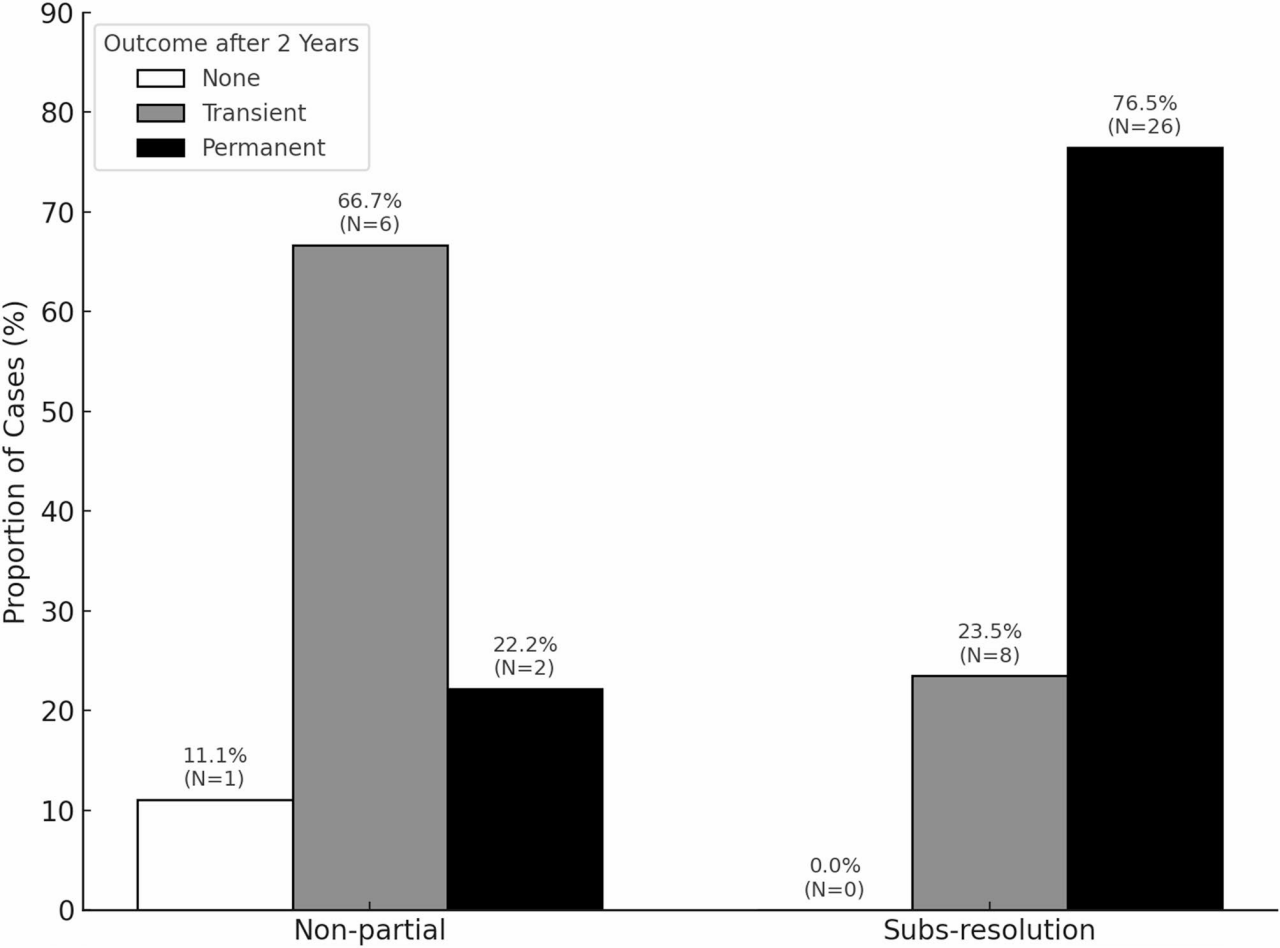
Long-term clinical outcomes (≥ 2 years) by T_6_ classification

Long-term outcomes (≥ 2 years) were evaluated in 43 cats from the refractory stomatitis cohorts. Among cats classified as “Subs-resolution” at T_6_, 76.5% (26/34) maintained permanent remission, and 23.5% (8/34) showed transient improvement. In contrast, cats classified as “non-partial” predominantly exhibited transient improvement (66.7%, 6/9), followed by permanent remission (22.2%, 2/9) and no improvement (11.1%, 1/9). Logistic regression analysis demonstrated that cats in the “Subs-resolution” group at T_6_ were significantly more likely to sustain clinical improvement for two years or longer than those in the “non-partial” group (odds ratio 6.5, 95% CI 1.32–32.08, *p* = 0.022; Fig. 5).

The proportions of cats in each T_6_ classification group including the “Non-partial” (No response and Partial improvement) and “Subs-resolution” (Substantial improvement to resolution) subcategories according to long-term outcome categories (permanent remission, transient improvement, and no improvement) are shown. Cats classified in the “Subs-resolution” group at T_6_ were significantly more likely to maintain clinical improvement for ≥ 2 years than those in the “non-partial” group (*p* = 0.022; odds ratio = 6.5).

## Discussion

The key to treating FCGS lies in the improvement of oral lesions, such as erythema, ulceration, and proliferation, in the mucosa of the oral cavity. Determining the underlying cause of FCGS continues to be an active area of study. Current etiologic theories for FCGS suggest a multifactorial pathogenesis involving a CD8 + T-cell-mediated immune response [17], persistent infection with FCV, upregulation of IL-6, IL-17, and disruption of the oral microbiome balance (dysbiosis) [15, 27–31]. The current standard of care is extracting all the teeth or all the premolar and molar teeth. Although this approach yields clinical improvement in 60% to 80% of cases—defined as complete resolution or a significant reduction of lesions with only mild to moderate residual inflammation—FCGS remains a unresolved condition that is often prone to relapse in some cases [9, 10, 32]. Due to these limitations, treatments such as stem cells, antiviral agents, and immunosuppressive therapies have been explored.

As described above, there are various medical treatments for recurrent FCGS after tooth extraction. Among these, a method using feline MSCs has recently attracted considerable interest. Several studies have documented the effect of mesenchymal feline MSC therapy for refractory cases of FCGS [8, 24–26]. However, there are no commercialized feline MSCs in Korea, and each veterinary hospital must directly collect tissues from healthy donors and isolate MSCs. This approach carries limitations, including difficulty in ensuring consistent cell quality and a potential risk of infection. In addition to MSC therapy, corticosteroids have also been investigated. Clinical improvement has also been observed with the use of corticosteroids in previously reported studies [10, 22]. However, due to their well-documented side effects, corticosteroids are generally not considered a viable long-term treatment option. Consequently, CsA or IFN may be regarded as treatment alternatives for refractory FCGS. CsA and IFN have both been utilized separately in refractory FCGS and have demonstrated clinical improvement. The therapeutic efficacy of the combination of these two medicines has not been examined. This study assessed the therapeutic efficacy of IFN and CsA in managing refractory FCGS by examining treatment outcomes and alterations in SDAI scores.

This study found that surgical treatment alone led to complete remission or significant improvement in 39.2% (47/120) of cases without the need for immunomodulatory therapy. This rate was significantly lower than those in prior reports [10, 11, 22]. This difference is believed to result from the restricted medical care in our protocol, which mainly included a 2-week regimen of antibiotics and analgesics post-dental extractions, with the use of supplementary drugs limited to analgesics alone.

Next, we administered IFN and CsA to treat refractory FCGS for a minimum duration of three months. In the refractory FCGS cases treated with IFN/CsA, 52.1% (38/73 cats) experienced clinical improvement (significant improvement or resolution) at the T_6_ timepoint. The efficacy of immunomodulatory agents, including CsA, IFN, and MSCs, for refractory FCGS has been documented in several studies [8, 11, 21, 22, 24, 26]. Previous reports have shown clinical remission rates of 45.5% with CsA over 3 months [21], and topical application of IFN to oral lesions once daily has been found to lead to clinical improvement in 55% (12/22) of the cases [22]. MSC therapy has shown the highest long-term remission rates, ranging from 57.1% to 71.4% depending on the source of MSCs [8, 24, 26]. In comparison, our study showed a 52.1% remission rate among refractory cases. Although lower than those reported for stem cell therapy, this rate is comparable to those observed in previous CsA- or IFN-based studies. This may be attributed to the consistent and prolonged (>3 months) administration of immunomodulatory therapy to all refractory cases.

In addition to evaluating clinical efficacy, we also monitored the occurrence of systemic adverse effects associated with immunomodulatory treatment using CsA and IFN (Table 4). The proportions of renal disease (8.2%) and cardiac disease (6.8%) appeared relatively higher; however, when compared with the prevalence reported in the general population, no substantial differences were identified. These findings suggest that the likelihood of these systemic complications being directly attributable to immunomodulatory treatment is low.

**Table 4:** Long-term evaluation of systemic safety following Immunomodulatory treatment

Affliction	Cases, *n* (%)^a^	Average time of onset (months)	Prevalence in general population (%)^b^
Anemia	2 (2.7)	28 (Range 8–48)	3.60
Renal disease	6 (8.2)	15.2 (Range 6–36)	23.10
Cardiac disease	5 (6.8)	21.6 (Range 8–48)	4.30
Gastrointestinal disease	2 (2.7)	13.5 (Range 6–21)	2.00
Hepatobiliary disease	2 (2.7)	10 (Range 8–12)	7.02
Diabetes mellitus	1 (1.4)	2 (Range 2)	0.08–1.24

ᵃ Values are expressed as n (% of total cases, *N* = 73)

ᵇ Prevalence in general population is based on previously published data (33–37)

In our investigation, 43 cats were available for long-term follow-up (2–6 years); of these, 65.1% (28/43) exhibited sustained clinical improvement. The increased improvement rate observed during long-term follow-up compared with the T_6_ timepoint indicates the potential for continued treatment interventions during the prolonged observation period. In research involving long-term monitoring after MSC therapy [8], the maintained improvement rate (2–9 years) was 58.6% (17/29; 9/17 autologous, 8/17 allogeneic), which is comparable to the results found in the present study with IFN/CsA.

A primary objective in the treatment of FCGS is to mitigate oral mucosal irritation. While the immune cell populations implicated in FCGS remain inadequately elucidated, numerous investigations have delineated distinct inflammatory patterns. Erythema of the posterior oral mucosa signifies an inflammatory response characterized by a substantial presence of lymphocytes and plasma cells inside the mucosal tissue. In particular, CD8 + T lymphocytes are recognized as increasing in both the bloodstream and lesion locations in felines with FCGS. Recent investigations have classified these cells into effector, central memory, and exhausted phenotypes, indicating functional variety within the CD8 + population. A predominance of effector and effector memory CD8 + T lymphocytes, coupled with a decrease in central memory subsets, has been linked to sustained mucosal inflammation and viral antigen stimulation [7, 8, 23]. These data suggest that, in addition to their prevalence, the functional status of CD8 + T cells may significantly contribute to the chronic immunological activation seen in FCGS.

IL-6 is another key cytokine in chronic FCGS, potentially increasing susceptibility to persistent viral infections such as FCV or FFV (FFV) infections that are directly associated with persistent inflammation and ulceration of the oral mucosa [7]. Whether the disease results from continuous antigenic stimulation or a dysregulated immune response remains uncertain, but both IL-6 overexpression and altered CD8⁺ T cell profiles appear to contribute to ongoing inflammation [7, 18].

The current investigation demonstrated that the combination therapy of CsA and IFN effectively managed the chronic erythematous inflammation present in the caudal oral mucosa in the long term. This indicates that both medications may play a role in moderating the inflammatory response linked to FCGS. Prior studies have indicated that IFN injection diminishes IL-6 levels, perhaps influencing FCV infection, a recognized factor in the pathophysiology of FCGS [38]. This mechanism facilitates the therapeutic efficacy of subcutaneous IFN treatment in ameliorating oral lesions in felines with FCGS [39]. CsA has demonstrated the ability to decrease CD8⁺ T cells [40, 41] and limit IL-17 production from memory Th17 cells [42], both of which are associated with the clinical signs of FCGS. Consequently, it can be inferred that the therapeutic efficacy of the CsA and IFN combination in this study may stem from the inhibition of CD8⁺ T-cell activity and the upregulation of IL-6. However, CsA, an immunomodulatory medication for refractory FCGS, should be used with caution due to its immunosuppressive properties. The prolonged administration of CsA has been linked to detrimental effects including hepatotoxicity, nephrotoxicity, anemia, and an elevated risk of toxoplasmosis [43, 44]. Special caution is necessary for cats with outdoor access or those consuming raw meat, as they may be at an increased risk for opportunistic infections.

Next, we evaluated the SDAI scores during the T_0_–T_6_ period for each group. At T_0_, the baseline SDAI scores were similar across all groups except that Group A exhibited a considerably lower SDAI score than Group C (*p* < 0.01). The findings indicate that the baseline (pre-extraction) SDAI score alone is inadequate for predicting long-term prognosis or categorizing future outcome groups in chronic FCGS cases.

At T_1_, Group A showed substantially lower SDAI scores than other groups, which was expected given that these cats achieved remission after tooth extraction with a single submucosal injection of IFN. Still, calculating ROC curve-based cutoff values offers a way to describe this distinction more objectively. The thresholds identified here (6.5 at T_1_ and − 14.5 for the T_0_–T_1_ change) should not be regarded as fixed clinical criteria, but they illustrate how the SDAI scores could be applied as a classification tool. Larger studies will be needed to determine whether such values can be refined into practical guidelines; however, our findings suggest that the SDAI scores may provide a useful reference when assessing prognosis in FCGS.

Beginning at T_1_, cats in Groups B, C, and D were classified as refractory cases and uniformly underwent a 3-month regimen of IFN/CsA therapy. At T_4_ (post-treatment), Group B had markedly lower SDAI scores than Groups C and D. The distinction remained statistically noteworthy until the T_6_ timepoint. While both Group B and Group C exhibited responses to IFN/CsA treatment, the SDAI scores for Group B were markedly lower than those of Group C starting at T_3_. The delayed divergence, occurring at T_3_ instead of T_2_ (one-month post-initiation of medication), may indicate the pharmacodynamic properties of CsA. CsA necessitates a consistent blood concentration over time, and it is likely that a duration exceeding one month is required for observable mucosal enhancement. In this study, Group D, which exhibited no clinical improvement after immunomodulatory therapy, constituted merely 1.7% of all cases. This discovery suggests that true non-responders to IFN/CsA are infrequent. The SDAI score decreased by 82% from T_1_ (the starting value) to T_6_ for all 73 cats that were treated with IFN/CsA. This indicated a marked clinical improvement.

Furthermore, in the refractory FCGS cohorts (Groups B, C, and D), long-term outcomes (2–6 years) were subsequently examined in cats accessible for prolonged follow-up. In this study, the clinical condition assessed at the T_6_ timepoint was found to be strongly associated with the likelihood of maintaining long-term remission. Logistic regression analysis indicated that cats categorized as exhibiting substantial improvement or resolution at the T_6_ timepoint possessed a significantly elevated probability of sustaining clinical improvement for two years or longer. This study indicates that clinical state evaluated at six months following the conclusion of three months of IFN/CsA therapy (i.e., at T_6_) is a substantial predictor of the probability of maintaining long-term remission.

The SDAI scoring system is a prevalent instrument in veterinary dentistry for evaluating treatment efficacy in FCGS, grounded in clinical manifestations and lesion severity [8, 45, 46]. Although the SDAI entails some subjectivity from the pet owner and may have constraints in accurately evaluating lesion severity, it has practical benefits regarding simplicity and immediacy. This tool is especially beneficial for assessing the initial response to immunomodulatory therapy in refractory FCGS cases.

The results of this study reinforce the therapeutic applicability of SDAI, showing a substantial association between SDAI scores and both short-term and long-term therapy outcomes. SDAI scores at the T_6_ timepoint were substantially correlated with clinical stability sustained over a period of two to six years. Consequently, SDAI may function as a pragmatic and dependable prognostic marker for long-term results. The findings indicate that assessing SDAI scores within a specified timeframe after immunomodulatory therapy can yield significant clinical insights, aiding in future treatment planning and prognosis conversations with pet owners.

The limitations of this study are as follows. First, it remains unclear how CsA and IFN exert their therapeutic effects in FCGS, making it difficult to fully understand their mechanisms of action in this condition. Second, if blood concentrations of CsA had been monitored across all individuals throughout the entire treatment period, it would have been highly valuable for interpreting the results of this study. Such monitoring could have enabled evaluation of whether differences in therapeutic CsA levels existed among Groups B, C, and D and might have explained the lack of clinical response in Group D if subtherapeutic CsA levels were present. Third, supportive medications used in this study did not include antibiotics or corticosteroids, which are commonly administered in other studies. Thus, direct comparisons with previous findings may be constrained.

In summary, this study highlights two key aspects. First, the combination of IFN and CsA may serve as a viable therapeutic option for refractory FCGS, alongside MSC therapy, offering potential for sustained long-term improvement. Second, assessments performed six months after the completion of three months of immunomodulatory treatment can provide a reasonable estimate of a patient’s long-term prognosis over several years. These findings offer new insights into the clinical management of refractory FCGS and may contribute to more personalized therapeutic strategies. Although a definitive cure remains elusive, we hope this study represents a meaningful step toward improving patient outcomes and guiding future research.

## Conclusions

This study evaluated the long-term effectiveness of combined IFN and CsA therapy in refractory FCGS. More than half of the cats achieved clinical improvement, and 76.5% of those who showed improvement at 6 months after 3-months of immunomodulatory therapy maintained remission for 2 to 6 years. These results suggest that combined IFN and CsA therapy may serve as an alternative treatment option when other approaches, such as stem cells or steroids, are not feasible. In addition, evaluation at specific time point may help predict the long-term prognosis in refractory FCGS.

## Supplementary Information

Supplementary Material 1

## Data Availability

All data generated or analyzed during this study are included in this published article. Further inquiries can be directed to the corresponding author.

## Abbreviations

FCGS: Feline chronic gingivostomatitis
SDAI: Stomatitis disease activity index
CsA: Cyclosporine
IFN: Interferon-ω
EMM: Extended medical management
