# Supplementary material for: Long-term efficacy of cyclosporine and interferon-ω in feline chronic gingivostomatitis: insights from SDAI scores
Source: BMC Vet Res. 2025 Dec 16;21:708. doi: 10.1186/s12917-025-05141-9 (PMC12709781; doi:10.1186/s12917-025-05141-9)
Supplement: Supplementary file 1 — Supplementary Material 1 [file 12917_2025_5141_MOESM1_ESM.docx]

**
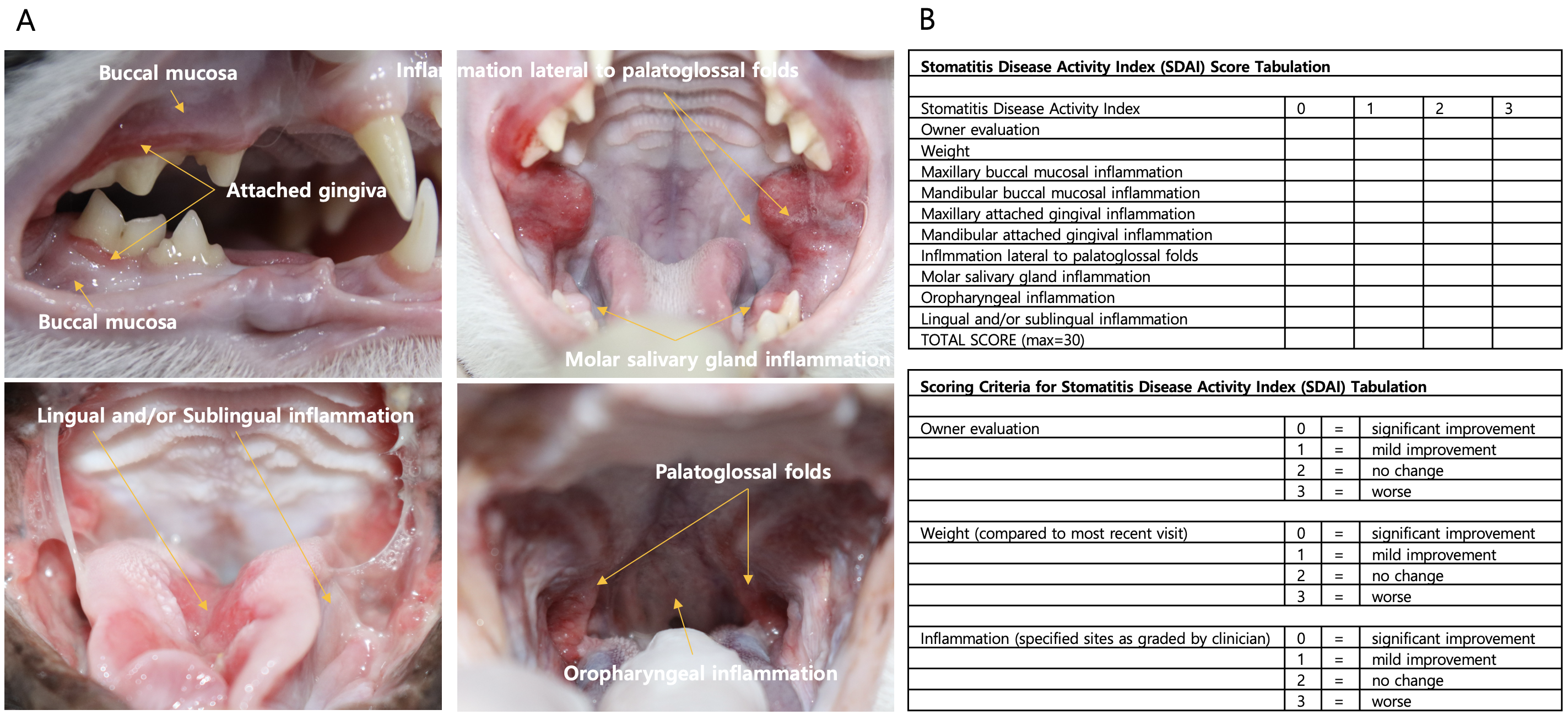
**

**Figure S1. Representative inflammation sites and tabulation scheme for the Stomatitis Disease Activity Index (SDAI).**

**(A)** Images show inflammation of the buccal mucosa, attached gingiva, molar salivary gland, palatoglossal folds, oropharyngeal, and lingual/sublingual areas used for scoring.

**(B)** The right panel summarizes the 0–3 scoring criteria applied to each component (0 = significant improvement, 1 = mild improvement, 2 = no change, 3 = worse).
